# Microbiological Assessment of White Button Mushrooms with an Edible Film Coating

**DOI:** 10.3390/foods12163061

**Published:** 2023-08-15

**Authors:** Margarida Machado Borges, Ana Sofia Simões, Carla Miranda, Hélia Sales, Rita Pontes, João Nunes

**Affiliations:** 1 Association BLC3—Technology and Innovation Campus, Centre Bio R&D Unit, Rua Nossa Senhora da Conceição n2, 3405-155 Oliveira do Hospital, Portugal; ana.simoes@blc3.pt (A.S.S.); carla.miranda@blc3.pt (C.M.); helia.sales@blc3.pt (H.S.); rita.pontes@blc3.pt (R.P.); joao.nunes@blc3.pt (J.N.); 2 BLC3 Evolution Lda, Rua Nossa Senhora da Conceição n2, 3405-155 Oliveira do Hospital, Portugal

**Keywords:** edible coatings and films, food microbiology, food preservation, microbial analysis, mushrooms, shelf life, food waste, circular bioeconomy

## Abstract

The development of edible coatings incorporating bioextracts from mushrooms native to Portuguese forests aims to enhance the value of the endogenous forest and mycological resources by harnessing their potential as a source of antimicrobial and antioxidant compounds. Edible coatings represent an important pathway to decreasing food waste and contributing to implementing a circular bioeconomy. The coating should result in product valorization through improved preservation/conservation, increased shelf life, as well as enhancement of its antioxidant and enzymatic properties. To evaluate the effectiveness of an edible coating on fungal food matrices, a 14-day shelf-life study was conducted, wherein both coated and untreated mushrooms were examined under controlled storage temperatures of 4 °C and 9.3 °C. *Agaricus bisporus* was chosen as the food matrix for its bioeconomy significance, and *Pleurotus eryngii* was selected for the preparation of the food-based coating due to its profile of bioactive compounds. Microbiological analysis and physicochemical monitoring were conducted on the food matrices and the coating. Coated mushrooms had less mass loss and color change, and had better texture after 14 days. Microbiological analysis revealed that the coating had no antimicrobial activity. Overall, the coating improved the shelf life of the coated mushrooms but had less effect on the microbial community.

## 1. Introduction

Currently, the increase in shelf life, the significant reduction of non-natural preservatives in the context of the clean label market, and the consumer demand for healthier foods are some of the main challenges in the food industry [1]. This means that food preservation technologies are now facing significant challenges to fulfil two additional objectives: ensuring the suitability of the processes used and generating environmentally friendly products [2]. The food industry is tasked with offering consumers fresh, pleasant, high-quality food with beneficial health properties. However, meeting consumer demands is challenging because no food can maintain its optimal conditions and properties indefinitely due to natural deterioration caused by chemical and biochemical reactions, and physical changes [3].

The use of natural bioactive ingredients and edible coatings has proven to be a technology with great potential to achieve longer shelf life while simultaneously ensuring food safety and quality attributes [4,5]. Bioactive components are constituents found in small amounts in food that can have health effects upon consumption. These components can display multiple properties simultaneously, potentially producing a synergistic effect [5,6]. An edible film or coating is a material with a thickness of less than 0.3 mm, composed of biopolymers and various additives dispersed in aqueous media. Edible coatings are applied directly to the food, while edible films are pre-made and then adhered to the product. These coatings and films possess several key characteristics, including protection against UV light, transportation of solutes, water vapor, organic vapors, and gases between the food and the atmosphere, mechanical damage resistance, increased shelf life, the presence of bioactive components, antimicrobial properties against bacterial reproduction and fungal contamination, beneficial microorganisms for the consumer, and use of biodegradable natural materials [2].

While many characteristics of edible films and coatings are relevant, the biological protection of food is particularly crucial as it directly impacts the product’s shelf life. Therefore, it is necessary to inhibit or eliminate bacterial or fungal microorganisms, along with their enzymes and byproducts, which can cause or accelerate food spoilage [2,7]. Oxygen availability, temperature, relative humidity, water content, and pH influence food spoilage. Thus, in addition to the characteristics of edible films, preserving microbiological parameters in accordance with established regulations, maintaining nutritional content, and preserving physical and sensory characteristics (such as smell, taste, and texture) are of great interest [8].

Current research trends involving active packaging alternatives focus on reducing oil consumption in deep-fat fried products, transporting bioactive compounds, and extending the shelf life of highly perishable items [8]. The commercial use of edible films has been limited due to issues related to their inferior mechanical and barrier properties compared to synthetic polymers [9]. In Europe, the European framework regulation (2004/1935/EC) allowed for the concept of active packaging with the intentional release of active agents. Regulation 1935/2004/EC repealed this legislation to facilitate technological innovation in packaging [7]. Consequently, these market demands have led to the need for alternative compounds for food applications, either as direct preservatives or applied in coatings that deactivate deteriorating reactions in food, while still ensuring the expected quality attributes for consumers [10]. The compounds that constitute the preservatives and coatings must meet specific requirements, including being of natural origin, being renewable, edible, and ideally, providing additional bioactive nutraceutical properties [11].

From the perspective of a circular and sustainable economy, which aims to increase process yields by reducing or even eliminating byproducts and waste while ensuring quality and safety in the final products, the creation of new products that value the byproducts and waste of a process, sometimes even reducing time-consuming and costly processes, establishes a cycle where everything is reused [12]. In this context, the utilization of byproducts and rejects from native Portuguese mushroom species, produced in closed systems (using agroforestry substrates) as a source of antimicrobial and antioxidant compounds for application in food preservatives and coatings, presents an excellent opportunity for high-added value products. In fact, the mycological food industry generates significant quantities of surpluses, rejects, and byproducts with each production cycle. The use of these products resulting from the production process and the elimination of processes that would otherwise lead to their extinction emerges as a productive advantage [12].

Mushrooms native to Portuguese Funga [13] have been the target of several studies to determine their bioactive compound profiles, as is the case of *Pleurotus eryngii*. *P. eryngii*, commonly known as the King Oyster Mushroom, is a saprobic mushroom with a wide geographic distribution, spanning from northern Africa to central Asia [14]. This edible macrofungus can be found growing on dead wood in humid seasons [15]. *P. eryngii* mushrooms offer a wealth of nutrients, boasting ample proteins, vitamins, and minerals, while maintaining a low-fat content. As a result, they prove to be an excellent choice for those dealing with hypertension, high blood low-density lipoprotein (LDL) cholesterol, obesity, metabolic disorders, and diabetes. Moreover, these mushrooms boast an array of advantageous bioactive compounds, including β-glucans, renowned for their antitumor, antioxidant, and immunomodulating capabilities. Additionally, *Pleurotus* mushrooms contain ergosterol, a precursor to vitamin D2 found in fungal cell membranes [16]. Ergosterol, a sterol whose structure is similar to cholesterol, seems to display hypocholesterolemic activity by interfering with cholesterol adsorption during digestion [15]. Polyphenols and flavonoids extracted from this species showed good antioxidant [17] and anti-inflammatory [18] activity. Polyphenols also demonstrate cytotoxic properties [18]. The wide range of bioactivity potential of *P. eryngii* makes it particularly interesting for the development of bioactive packaging alternatives.

Shelf-life studies can be used to determine the effectiveness of the application of an edible coating in foodstuffs. The white button mushroom (*Agaricus bisporus*) was selected as the food matrix for this shelf-life study as it holds the distinction of being the most extensively cultivated and consumed mushroom globally, accounting for approximately 40% of total worldwide mushroom production [19]. Targeting microbiological assessments and physicochemical changes is usually used to determine food quality, as well as storage effectiveness [20]. Selecting the most relevant parameters for the formulated coating can help in determining its usability.

Microbiological analysis is one of the most relevant parameters in a shelf-life study [21]. The values of the overall number of microorganisms present in a milliliter or gram of sample and capable of growing at 30 °C can give us an estimate of the microbial biomass contained in the sample. These values can help us quantify the potential inhibitory effect that both the developed coating and storage temperature have on microbial growth. Molds and yeasts are the main microorganisms responsible for the degradation and decomposition of food, so their enumeration can indicate the viability of food matrix storage and their state of preservation [22]. Their values also allow us to quantify the potential beneficial effect of both coating application and storage temperature. The counting of *Escherichia coli* can detect and quantify this species, thus contributing to the microbiological characterization of the food matrix used. *E. coli* is a bacterial species present in the human microbiome and is not considered pathogenic. However, it can behave as an opportunistic pathogen in certain situations [22]. Moreover, this bacterium is a member of the Enterobacteriaceae family whose presence in foods most consistently indicates potential contamination with animal fecal material, although its presence is not necessarily associated with the presence of enteropathogens [23]. The Enterobacteriaceae family comprises several species of pathogenic bacteria that can alter the composition of food by releasing toxins (enterotoxins) into the food where they grow, thus contaminating it [22]. This family characterization serves as an indicator of the coating’s action and the incubation temperature on this group of bacteria. Coagulase-positive *Staphylococcus* characterization and count help determine the level of contamination of food matrices with this group. The genus *Staphylococcus* is composed of some pathogenic species, most of which are highly sensitive to thermal processing and disinfection by chemical agents. Therefore, their presence indicates poor hygiene conditions in the handling of food matrices [22]. These bacteria can survive on fresh mushrooms, and the conditions of low oxygen and high carbon dioxide inside unventilated polyvinyl chloride (PVC) packages favor the growth of facultative anaerobic microorganisms such as them [24]. This bacteria analysis allows for the determination of whether mushrooms are safe for human consumption and whether this group of bacteria is affected by the presence of the applied edible film. *Bacillus cereus* quantification allows for determining if the mushroom is contaminated with this species and estimating its level of contamination since it is very common for this species to be detected in very high quantities [22]. This analysis allows for determining whether the mushrooms are safe for human consumption and whether this bacterial species is affected by the application of the edible coating. *Pseudomonas* quantification allows the characterization of the population of microorganisms present in food and determines their state of preservation. This group of organisms is commonly found in fungal food matrices and is one of the most evaluated target groups in shelf-life studies [25]. In this case, the procedure was also used to compare the results of coated matrices with the control, as well as the results of two storage temperatures. The purpose of *Salmonella* spp. detection is to identify the presence of this bacterial genus, since it can have a pathogenic effect even in very small quantities when present in food [22]. The procedure helped in characterizing the microbial populations present in mushrooms and their reaction to the developed edible film, as well as to the storage temperature. *Salmonella* spp. detection is of particular importance in this work as these organisms are included in the legislation governing food safety for foods such as mushrooms [26]. The purpose of *Listeria monocytogenes* detection is to identify the presence of this bacterium in food matrix samples, as its presence can cause listeriosis. *L. monocytogenes* is the only species within the genus *Listeria* that is pathogenic to humans, and its detection in a food matrix is considered serious contamination [22]. This species is mentioned in the regulation that establishes which organisms are relevant in this type of food [26].

Physicochemical alterations throughout the shelf-life study can be used to determine the efficacy of the edible coating. Parameters like pH, weight loss, color, and texture [27] can be monitored at the start time and after the incubation period. The differences between measurements allow for the determination of a positive effect of applying an edible coating. Monitoring pH can be used to determine good food preservation. Food spoilage, especially when caused by bacteria, can change the pH. By detecting these changes, it is possible to evaluate the effectiveness of food storage techniques. Additionally, by determining the edible coating’s pH, potential antimicrobial effects can be inferred. It is beneficial to have pH in consideration when formulating a new edible coating, as low pH can be used to modulate microbial growth since these organisms usually do not respond well to acidic environments [2]. Weight loss is a crucial parameter as it can be directly translated into an economic loss [28]. Moreover, it is a common phenomenon during food stuff’s storage, mainly due to loss of water during the respiration processes [7]. Mushrooms’ structure fails to prevent excessive moisture loss and therefore they not only have a very high transpiration rate but their appearance is strongly affected by this process [28]. Registering weight loss data during shelf-life studies provides information on the effectiveness of the applied coating in preserving the visual and physical characteristics of the food matrices [7]. Color is a highly variable parameter. However, changes in color between coated and uncoated matrices can determine the commerciality of the developed coating. Moreover, changes in color over time can be indicative of the oxidation processes association with food degradation [2]. Food texture is an important parameter, as changes in texture can indicate a loss of quality. Increases in firmness indicate water loss, and limpness points to microbial degradation. An edible film coating intends to preserve or improve food matrix textures, making the final product more desirable [2].

The objective of the present study was to evaluate the efficacy of an edible food coating with the incorporation of extracts and bioactive compounds from *P. eryngii*. The purpose of this coating was to enhance the value of the product onto which it was applied by improving its preservation, extending its shelf-life time, and enhancing its antioxidant and enzymatic properties. To assess the efficacy of the edible food coating on a fungal food matrix, both coated mushrooms and the control underwent a shelf-life study, which included their storage at two controlled temperatures for 14 days.

## 2. Materials and Methods

### 2.1. Edible Film Coating

In order to produce edible film coatings, byproducts and non-compliant products of *Pleurotus eryngii* production were used to obtain aqueous extracts with a high content of polysaccharides using a heat-assisted procedure based on the method described by Aguiló-Aguayo et al. [29] and later established by Taofiq et al. [15]. The edible film coating was then produced according to the procedure adapted from Odila Pereira et al. [30]. The obtained aqueous and lyophilized extract, rich in bioactive compounds, was used in addition to citric acid (a preservative) and glycerol (as a plasticizer).

### 2.2. Application of the Edible Film Coating on Mushrooms

After its production, and through immersion, the edible film coating was applied to *Agaricus bisporus* mushrooms. The mushrooms remained immersed until the coating completely covered them, thus establishing a complete film layer. As a control group, from the same batch, mushrooms were immersed in sterile distilled water. Both samples were left to air dry for a few hours until they were completely dry to the touch [5,8].

### 2.3. Shelf-Life Study

Immediately after the coating application, samples were stored at a controlled temperature without air circulation for 14 days, the maximum reported shelf life at controlled temperatures [31]. The selected storage temperatures were 4 °C, following the European Food Safety Authority’s recommended consumer-level refrigerator temperature, and 9.3 °C, which corresponds to the highest temperature documented in survey data from domestic refrigerators in Portugal [21,32].

#### 2.3.1. Microbiological Analysis

During the shelf-life study, in accordance with Schill et al. [33], samples for microbiological analysis were collected from both the coated and control mushrooms at the study’s outset (day 0, t_0_) and conclusion (day 14, t_14_). Additionally, a sample of the coating in its liquid form was collected before drying. To start the procedure, an initial solution of 100 g/L and sequential serial dilutions (from 100 g/L to 0.01 g/L) were prepared from the initial samples in Buffered Peptone Water (VWR International, Geldenaaksebaan, Leuven, Belgium), following a protocol adapted from ISO (International Standardization Organization) 7218:2007.

The total microorganism count at 30 °C was carried out in accordance with NP (Norma Portuguesa) 4405:2002 to determine the number of colony-forming units present in a gram of sample (log CFU/g), capable of growing at 30 °C in Plate Count Agar culture medium (VWR International, Geldenaaksebaan, Leuven, Belgium).

The counting of molds and yeasts was carried out in accordance with ISO 21527-1:2008 to detect and enumerate these organisms’ growth in log CFU/g using Rose-Bengal Chloramphenicol Agar culture medium (Oxoid Ltd., Basingstoke, Hampshire, England).

*Escherichia coli* count was conducted in accordance with NP 4137:1991 to detect and enumerate this species in log CFU/g using Tryptone Bile Glucuronide culture medium (VWR International, Geldenaaksebaan, Leuven, Belgium).

Enterobacteriaceae count, adapted from NP 4137:1991, was carried out to detect and enumerate, in log CFU/g, bacteria belonging to this family through enrichment in Milk Agar culture medium and enumeration in Violet Red Bile Glucose Agar culture medium (Oxoid Ltd., Basingstoke, Hampshire, England). A confirmation step with Nutrient Agar culture medium (PanReac Química S.L.U., Barcelona, Spain), oxidase reagent and Oxidative-Fermentative Agar culture medium ensured that only target bacteria were counted.

Coagulase-positive *Staphylococcus* count was performed in accordance with ISO 6888-1:1999 to detect and enumerate this species in log CFU/g using Baird Parker culture medium (VWR International, Geldenaaksebaan, Leuven, Belgium). To ensure the accurate counting of target bacteria, a confirmation step was conducted using Brain Heart Infusion culture medium (VWR International, Geldenaaksebaan, Leuven, Belgium) and a rabbit plasma coagulation test.

*Bacillus cereus* count, according to ISO 21871:2006, was carried out to detect and enumerate this species in log CFU/g using Tryptone Soy Broth culture medium supplemented with Polymyxin B (PanReac Química S.L.U., Barcelona, Spain) in an enrichment step and using Mannitol Egg Yolk Polymyxin agar culture medium (VWR International, Geldenaaksebaan, Leuven, Belgium) in the enumeration step. To ensure the accurate counting of target bacteria, Blood Agar culture medium (E&O Laboratories Limited, Bonnybridge, Scotland) was used in a confirmation step.

The quantification of *Pseudomonas* was performed according to ISO 13720:2010 to characterize this population of microorganisms using *Pseudomonas* agar culture medium (Scharlab, S.L., Barcelona, Spain). To ensure the accurate characterization of the target population, a confirmation step with oxidase reagent was carried out.

*Salmonella* spp. detection was conducted according to ISO 6579-1:2017 to identify, confirm and characterize this bacterial genus with enrichment in Buffered Peptone Water (VWR International, Geldenaaksebaan, Leuven, Belgium) and Rappaport-Vassiliadis-Soya Broth culture medium (VWR International, Geldenaaksebaan, Leuven, Belgium), detection in Deoxycholate-Lysine-Xylose Agar culture medium (VWR International, Geldenaaksebaan, Leuven, Belgium), and screening in Nutrient Agar (PanReac Química S.L.U., Barcelona, Spain), Triple Sugar Iron Agar (VWR International, Geldenaaksebaan, Leuven, Belgium), Urea Agar (VWR International, Geldenaaksebaan, Leuven, Belgium) and L-Lysine decarboxylation broth (VWR International, Geldenaaksebaan, Leuven, Belgium) culture media.

*Listeria monocytogenes* detection was executed according to ISO 11290-1:2017 to identify and confirm the presence of this bacterium with enrichment in Half-Fraser and Fraser broth culture media, detection in Ottaviani and Agosti Agar (VWR International, Geldenaaksebaan, Leuven, Belgium) and Violet Red Bile Glucose Agar (Oxoid Ltd., Basingstoke, Hampshire, England) culture media, and confirmation in Blood Agar (E&O Laboratories Limited, Bonnybridge, Scotland), Rhamnose broth (Fisher Scientific Company, Fair Lawn, NJ, USA) and Xylose broth (Fisher Scientific Company, Fair Lawn, NJ, USA) culture media.

#### 2.3.2. Physicochemical Parameters

During the shelf-life study, samples were collected for the evaluation of the physicochemical parameters from both the coated and control mushrooms at the study’s outset (day 0, t_0_) and conclusion (day 14, t_14_).

##### pH

The pH value of the samples was determined through electrometry, following the procedure established by the Adolfo Lutz Institute with some modifications [34]. Triplicates from the collected samples were finely ground into powder and then mixed with deionized water at a concentration of 100 g/L through agitation for 5 min, followed by a resting period of 1 h. Afterwards, each sample was filtered, and the pH value of each one was measured using a pH meter. All the obtained values were duly recorded.

##### Weight Loss

Weight loss was determined by weighing the samples in triplicate at the study’s outset and conclusion. The differences between the final weight and initial weight were calculated and expressed as a percentage of weight loss using the equation:

(1) $$weight\;loss\;\left(\%\right)=\frac{W_{t0}-W{\;}_{t14}}{W{\;}_{t0}}\times 100;$$

where $W_{t0}$ is the sample weight at the study’s outset (day 0) and $W_{t14}$ is the same sample weight at the study’s conclusion (day 14) [25].

##### Color

The analysis of the triplicate samples’ cap color was facilitated through the use of a mobile phone application named “Color Grab”, based on research from the literature [35]. The color and luminosity system employed for this analysis was CIELCH(ab). From the application, three main parameters were provided: the “a*” value, characterizing coloration from red (+a*) to green (−a*); the “b*” value, characterizing coloration from yellow (+b*) to blue (−b*); the “L*” value, providing luminosity, ranging from white (L* = 100) to black (L* = 0) [36].

Additionally, ΔE*, corresponding to the comparison between samples, with and without coating, is obtained. A ΔE* > 2.3 indicates a noticeable difference in color perceived by the average human eye [36].

The necessary equation for ΔE* is:

(2) $$\Delta E^{*}=\sqrt{{{(L}^{*}{}_{2}-L^{*}{}_{1})}^{2}+{{(a}^{*}{}_{2}-a^{*}{}_{1})}^{2}+{{(b}^{*}{}_{2}-b^{*}{}_{1})}^{2}}.$$

##### Texture

At the outset (day 0) and conclusion (day 14) of the shelf-life study, the triplicate samples’ cap and stem textures were evaluated using Texture Profile Analysis (TPA) with a texture analyzer (CT3 Texture Analyzer, Brookfield Engineering Laboratories, Inc., Middleboro, MA, USA) and the Magness-Taylor cutting test. The weight of each sample was defined as the *Trigger.* The *Deformation* and *Test Velocity* were defined, respectively, as 1.4 mm and 2 mm^−1^. The percentages of *hardness* and *cohesiveness* variation between samples at the study’s outset and conclusion were calculated according to the equations:

(3) $$Hardness\;variation\;\left(\%\right)=\frac{Hardness{\;}_{t0}-Hardness{\;}_{t14}}{Hardness{\;}_{t0}}\times 100;$$

(4) $$Cohesiveness\;variation\;\left(\%\right)=\frac{Cohesiveness{\;}_{t0}-Cohesiveness{\;}_{t14}}{Cohesiveness{\;}_{t0}}\times 100;$$

where ${Hardness}_{t0}$ is the sample *hardness* at the study’s outset (day 0), ${Hardness}_{t14}$ is the same sample *hardness* at the study’s conclusion (day 14), ${Cohesiveness}_{t0}$ is the sample *cohesiveness* at the study’s outset (day 0), and ${Cohesiveness}_{t14}$ is the same sample *cohesiveness* at the study’s conclusion (day 14) [25].

### 2.4. Statistical Analysis

The obtained values for the microbiological analysis and physicochemical parameters, with at least two replicates for each condition, were all statistically evaluated and subsequently discussed considering the potential effect of the developed edible coating. *t*-tests, as well as Mann–Whitney, two-tailed, multiple comparison tests were applied to determine the difference among the means. The level of statistical significance was determined at 95% probability. The results from the statistical analysis are in the Supplementary Materials, Figures S1–S23.

## 3. Results and Discussion

### 3.1. Microbiological Analysis

Fresh edible mushrooms are included in the category of “Ready-to-eat foods—unprocessed and uncut fresh fruits and vegetables” in Regulation 2073/2005 of the European Commission. This legislation specifies that only the detection (and in some cases enumeration) of *Listeria monocytogenes* and *Salmonella* spp. should be performed since most other microorganisms can be eliminated during the preparation of the food for human consumption through heat processing. Although most of the protocols applied are not considered relevant in the regulation, they provide important data on the characterization of the microbial community in fungal food matrices, the effect of the applied coating and of storage temperature. In order to have a comprehensive view of the results, a group of organisms was considered detected when the confirmation tests were positive. According to the microbiological analysis conducted on both the coated and the control mushrooms, and in accordance with the parameters established by the applicable legislation [26], both were found to be safe for human consumption as no colonies of *Listeria monocytogenes* and *Salmonella* spp. were detected. However, other microorganisms were detected, and their results are relevant for the discussion on the efficacy of the edible coating. Table 1 and Table 2 summarize the obtained results.

When evaluating the tables above, it is possible to verify that total microorganisms at 30 °C, molds and yeasts, Enterobacteriaceae (only at t_14_ 9.3 °C) and *Pseudomonas* were detected. The groups coagulase-positive *Staphylococcus*, *Bacillus cereus*, and *Listeria monocytogenes* were initially detected but later identified as not belonging to the target species after confirmation tests.

#### 3.1.1. Total Microorganisms at 30 °C

These microorganisms were detected in all samples, regardless of the presence or absence of coating. Their enumeration varied between samples and time points. After statistical analysis (*t*-test), no significant differences in the application of coating or storage temperature were found between coated and control mushrooms.

At t_0_ 4 °C, control mushrooms had lower log CFU/g (5.85 ± 0.00 log CFU/g) than coated mushrooms (uncountable). At t_14_ 4 °C, the trend was maintained with coated mushrooms at uncountable and control mushrooms at 6.58 ± 0.00 log CFU/g. It is worth noting that high CFU counts, above 300 CFU per plate and considered uncountable, caused data gaps and affected mean and standard deviation. Log CFU/g increased proportionally with time for control mushrooms. It was impossible to determine the same for coated mushrooms as plates were uncountable since t_0_. For the storage temperature of 4 °C, the values for control mushrooms fall within the intervals reported in the literature [25], which states values between 5.5 and 7.5 log CFU/g. Coated mushrooms had counts higher than reported in the literature. Falguera et al. [7] reported that storage temperature can improve the antimicrobial effects of edible films. However, our data suggest an opposite effect of a 4 °C temperature for the developed coating, which resulted in coated mushrooms with unmeasurable CFU counts.

At 9.3 °C, at both sampling times, coated mushrooms had slightly higher log CFU/g (3.72 ± 0.89 log CFU/g for t_0_ and 6.02 ± 0.35 log CFU/g for t_14_) than control mushrooms (2.93 ± 0.21 log CFU/g for t_0_ and 5.55 ± 0.21 log CFU/g for t_14_). This trend is coherent with the data obtained for storage at 4 °C and Log/CFU values increased for both treatments over time. Coated mushrooms showing higher counts is an unexpected result, as the antimicrobial properties of the mushroom extract (rich in bioactive compounds) used in the edible coating formulation should inhibit microbial growth [8]. Both coated and control mushrooms fall within the CFU counts reported in the literature [25] for the storage temperature of 9.3 °C. t_0_ counts are slightly lower than those previously reported. For this storage temperature, the interaction between coating and temperature seems not to be as bad as for 4 °C. Coated mushrooms showed results with values within the expected intervals, which shows that the application of the coating does not hinder mushroom preservation.

The results for both storage temperatures seem to indicate that a higher temperature provides better results for total microorganisms at 30 °C enumeration. In future studies, it could be interesting to determine whether room temperature provides the same or better results for the application of the coating. Edible coatings can be applied to foods meant to be kept at room temperature [7], and reducing the need for the cold storage of mushrooms can be a more ecological approach for their storage. Additionally, it is important to note that the coating is not sterile (see Section 3.2), and the results being observed can be influenced by the microbial community inherently present in it.

#### 3.1.2. Molds and Yeasts

Molds and yeasts were detected in all samples, for both coated and control samples, at both sampling times. This analysis yielded varied results between sampling times. Regardless, the high number of uncountable plates had a significant influence on the obtained means. *t*-tests were performed to verify the influence of the coating and temperature, comparing the results obtained for coated and the control samples at each sampling time and the results obtained for t_0_ and t_14_ at both storage temperatures. Nevertheless, and unlike what was expected, none of the comparisons showed a significant influence of either the coating or the temperature on the results (the differences were not statistically relevant).

In t_0_ at 4 °C, control mushrooms had lower log CFU/g values (6.46 ± 0.00 log CFU/g) than coated mushrooms (uncountable). In t_14_ at 4 °C, control mushrooms still had lower counts (4.18 ± 0.16 log CFU/g) than coated mushrooms (6.02 ± 0.00 log CFU/g). It is noteworthy that CFU values decreased over time for both treatments. The decrease in CFU counts is unexpected for control mushrooms. However, this observation can be explained by the weight loss observed (see Section 3.3.2). Weight loss is mainly affected by water loss due to respiration [7]. Lower water contents can make for a more hostile environment for microorganisms and, therefore, justify lower counts after 14 days of incubation.

At t_0_ 9.3 °C, coated mushrooms had lower CFU counts (3.80 ± 0.50 log CFU/g) than control mushrooms (4.00 ± 0.14 log CFU/g). At t_14_ 9.3 °C, coated mushrooms had higher CFU counts (5.11 ± 0.10 Log/CFU) than control mushrooms (4.60 ± 0.00 log CFU/g). Over time, CFU values increased for both treatments. This inversion in trend and the lower CFU counts, when compared with the results for storage at 4 °C, seem to suggest the fact that temperature is a more determining factor in inhibiting the proliferation of these microorganisms.

The values obtained for both treatments in all storage temperatures are higher than those reported in the literature [23], which indicates log CFU/g at approximately 3.2–3.4 for control mushrooms. However, in the literature, the samples were transported to the laboratory in insulated boxes with ice packs, unlike these samples that were transported in produce delivery boxes at room temperature before the application of the coatings. Khan et al. [37] reported that transport temperature plays a vital role in mushroom shelf life, and therefore this should be taken into consideration in future approaches.

The results for both storage temperatures point to the fact that a higher temperature provides lower counts for molds and yeasts enumeration. In future studies, as previously mentioned, room temperature storage should be tested. As for molds and yeasts, it is important to note that the coating is not sterile (see Section 3.2), and the results being observed can be affected by the high log CFU/g found.

It can also be argued that inter-batch variability can also play a role in the obtained results, for both total microorganisms at 30 °C, and molds and yeasts enumeration. Batches were mixed before starting the shelf-life study. However, mushrooms stored at 9.3 °C seem to have provided better results overall. This temperature is not the ideal food preservation temperature and it can be postulated that it should have provided the worst results observed. In that case, batch quality can be more determining than storage temperature, and in turn, explain the observations.

#### 3.1.3. *Escherichia coli*

*Escherichia coli* was not detected in any of the samples at either t_0_ or t_14_, irrespective of the coating, temperature, or sampling time. However, given the nature of the protocol, it is possible that *E. coli* was present in the tested samples but in quantities below the detection limit of the procedure, requiring additional tests (e.g., molecular detection). Therefore, since no colonies were detected even in the control mushrooms, it is not possible to assess the effect of coating or storage temperature on the growth of this species. In the literature, similar results are reported. According to Venturini et al. [23], no mushrooms were contaminated with a virulent strain of *E. coli*. and Gónzalez-Fandos et al. [38] also reported the absence of *E. coli* in *A. bisporus*. In another study of *A. bisporus*, none of the 202 samples screened had this pathogen [39].

#### 3.1.4. Enterobacteriaceae

Enterobacteriaceae were detected in all samples, except for both samples in t_0_ of 9.3 °C. After confirmation tests, bacteria from this group were only identified at t_14_ at 9.3 °C. The samples in t_14_ at 9.3 °C had so much bacterial growth the plates were uncountable. This result may indicate a very high number of log CFU/g or colonies with very robust growth. In the remaining conditions, enumeration was obsolete as confirmation tests were negative. In future studies, it may be interesting to make additional dilutions of the initial sample to try to obtain countable replicas in this procedure.

Additionally, since bacteria from this group were only found at t_14_ at 9.3 °C, it may indicate a stimulating effect on the samples of the higher temperature on this group of bacteria over time. In the literature, blotch symptoms in mushrooms are often associated with bacteria from this family [40], and their presence often results in sensory deficits and higher microbial loads and influences the storage stability of the mushrooms [33]. Regardless, the applied coating is not effective in inhibiting the growth of this bacterial group as it had no effect in preventing its development in coated mushrooms.

#### 3.1.5. Coagulase-Positive *Staphylococcus*

Coagulase-positive *Staphylococcus* analysis allowed us to determine that both coated mushrooms and the control were not contaminated with this species at any of the sampling times. Initially, for a storage temperature of 9.3 °C, a significant number of suspicious colonies were detected, but confirmation tests revealed that they belonged to other taxa. Therefore, it was not possible to determine whether the coating would be effective in reducing this pathogen in food matrices. In the literature, one study isolated *Staphylococcus* spp. from 35 out of 156 mushroom samples, 22.4% of the total analyzed samples. However, only four isolates were later identified as *Staphylococcus aureus* [41]. In another study, similar to our results, *Staphylococcus* was not isolated from any sample [23].

#### 3.1.6. *Bacillus cereus*

*Bacillus cereus* was not present in any of the tested samples. At t_14_ and 4 °C, suspicious colonies were detected in only one dilution of the control mushroom sample, but confirmation tests determined that they did not belong to the target species of the study.

According to the literature, one of the most prevalent zoonotic agents in both dried and brined mushrooms was *B. cereus*; one study reported that 81.5% of dried mushroom samples they tested harbored enterotoxigenic *B. cereus* (≥5.0 log CFU/g) [42]. Another study, similar to our results, did not detect *B. cereus* in any mushroom sample [33]. Since no colonies of this bacterium were detected, we could not determine the effectiveness of the coating against it.

#### 3.1.7. *Pseudomonas*

*Pseudomonas* was detected and confirmed in all samples at both t_0_ and t_14_, regardless of the presence or absence of coating. However, it was not possible to count the colonies present in any of the dilutions, as individualized colonies could not be visualized in any of the tested dilutions. The fact that this bacterium was detected in all tested samples indicates that the coating is not efficient in inhibiting the growth of these bacteria. Furthermore, one study also found that Pseudomonadaceae were the most prevalent group, occurring in all mushroom species [23], and one other study confirms that one of the most abundant species across all of their mushroom samples was a *Pseudomonas* species (58.4%). This study also suggests that severe changes in the sensory quality were mainly observed in such stored samples where *Ewingella americana*, from the Enterobacteriaceae family, was found in combination with different *Pseudomonas* species [33]. Additionally, due to their prevalence, it can be inferred that these bacteria might be part of the microbiome of the used food matrices. Additional studies are needed to confirm this hypothesis or find a source of contamination in the production chain.

#### 3.1.8. *Salmonella* spp.

*Salmonella* spp. was not detected in any of the samples, regardless of the presence or absence of coating. The search for this species is of particular importance as it is required by the regulations applicable to fungal food matrices, and the absence of *Salmonella* spp. aligns with the values allowed by the legislation. It was concluded that the tested mushrooms were safe for human consumption in terms of this parameter. Still, the effectiveness of the coating against these microorganisms could not be determined as they were not detected. In the literature, similar results can be found. Two studies were also unable to isolate *Salmonella* spp. in cultivated mushrooms [23,33]. Nevertheless, one study detected this bacterium in 5% of fresh mushrooms commercialized in Seattle (USA) [43].

#### 3.1.9. *Listeria monocytogenes*

Both coated and control mushrooms were safe for human consumption according to the legislation, as no colonies of *Listeria monocytogenes* were detected in any of the tested samples. Initially, suspicious colonies were detected in all control mushroom samples (except for t_0_ at 9.3 °C) and in coated mushrooms at t_14_ and 9.3 °C. Confirmation tests revealed that these suspicious colonies belonged to other species within the same genus, which are not pathogenic to humans. Likewise, one study did not detect these bacteria in any of their samples. Even so, several other studies documented the presence of *L. monocytogenes* in either processed or wild mushrooms [23,44,45]. Moreover, mounting evidence suggests that *L. monocytogenes* contamination occurs during the processing stage, emphasizing the importance of monitoring the production chain from substrate production to harvest, processing and packaging [33]. Since no colonies of the target species were detected, it was not possible to evaluate the effect of the edible coating on it.

In general, when evaluating the effect of the edible coating with the incorporation of mushroom bioactive compounds on total organisms at 30 °C and mold and yeast counts, it was concluded that the edible coating did not have an effect in reducing the number of log CFU/g. Therefore, it would not be sufficient to reduce the degradation rate of mushrooms by these organisms over the shelf-life time. These results also suggest that the coating does not have antimicrobial effects, as its presence does not prevent or reduce the proliferation of microorganisms. This result was unexpected, as the presence of bioactive compounds in edible coatings applied to mushrooms usually results in the inhibition of microbial growth [46].

### 3.2. Microbiological Analysis of the Edible Coating

The microbiological analysis of the edible coating was performed using the same protocols applied to the food matrix samples. As a result, total organisms at 30 °C were detected (with all plates being uncountable), *Pseudomonas* (with replicas showing significant growth), and mold and yeast counts (with all plates being uncountable). These groups were also detected in both the coated and the control food matrices. This fact may indicate the absence of antimicrobial effects of the coating, as it does not prevent the proliferation of microorganisms even in the absence of a food matrix. It is also important to mention that the coating used incorporates bioactive compounds derived from a mushroom extract, so some of these organisms may appear in the coating as they are already part of the mushroom’s microbiome from which the extracts were obtained.

The edible coating was not produced in aseptic conditions, nor were the many components of the coating’s formulation sterilized. Due to this, we cannot expect a total absence of microbial organisms in a microbiological assay (on both the coating and the food matrix). Formulating an edible film coating in aseptic conditions can be advantageous for several reasons. Aseptic conditions ensure that the formulation process is free from microbial contamination. Microorganisms, such as bacteria and fungi, can compromise the safety and quality of the edible film coating. By working in a sterile environment, the risk of introducing harmful microbes into the formulation is minimized, reducing the potential for microbial growth and contamination of the final product. Secondly, edible film coatings are applied to perishable food products to extend their shelf life. If the formulation process is not carried out in aseptic conditions, there is a higher chance of introducing spoilage microorganisms, which could negate the intended preservation benefits of the coating. Aseptic work conditions can help maintain the integrity and effectiveness of the coating, preserving the quality and safety of the coated food product for a longer period [47]. However, aseptic conditions are not commonly used in industrial applications due to increasing costs and process engineering. Adding additional steps to ensure asepsis in the edible film coating’s manufacturing process can make this product non-viable. When creating an edible film to utilize it in the current markets it is necessary to take into consideration the production costs and time, as well as how it will affect the consumers’ opinion of the product. If the edible film coating becomes too expensive, consumers will opt for the traditional packaging options and this new technology will not integrate into market [48].

Regardless, it could also be important to ascertain the influence of sterilization of the coating’s components and aseptic work conditions in this type of microbiological study. Although the results demonstrated a lack of antimicrobial effect from the edible coating, there is no guarantee that the same would be observable under more sterile conditions. This could be an improvement to the application and manufacturing process of the coating in subsequent studies.

### 3.3. Physicochemical Parameters

#### 3.3.1. pH

As previously established, pH serves a crucial role in regulating food preservation and assessing the impact of coatings on the overall quality of mushrooms [2]. The pH values for both coated and control mushrooms, at the beginning and end of the shelf-life study for both storage temperatures, are listed in Table 3.

The pH values obtained for each sample fall within the range of 6.35 to 6.75. For both storage temperatures and both sampling times, the pH value is always slightly lower in the sample without coating, except for the 9.3 °C t_0_ condition where this trend is reversed. This overall trend might be explained by the fact that the applied bioactive coating contains citric acid. Regardless, according to the statistical analysis, no significant differences can be found between samples. Overall, these pH values are within the expected parameters and consistent with the literature. Research from Martinez-Carrera et al. indicates a low-acid pH, ranging from 5.7 to 6.8, for fresh mushrooms [49].

#### 3.3.2. Weight Loss

The reduction in weight of food samples is typically attributed to the loss of water through respiration processes and can directly affect profits. Mushrooms are particularly susceptible to this phenomenon; therefore, by monitoring weight loss data throughout the shelf-life study, we can gauge how effectively the applied coating maintains the food’s visual and physical attributes [7,28].

The results for weight loss determination for coated and control mushrooms are presented in Table 4.

Based on the information provided in Table 4, the shelf-life study at 4 °C shows similar weight losses in both samples, 83.35% in the control samples (without coating) and 81.96% in the coated samples.

At 9.3 °C, the results show the samples without coating with higher weight losses than the coated samples. The control samples have 87.43% of weight loss and the coated samples have 81.90%. These findings suggest that the bioactive coating potentially functions as a protective barrier, helping to reduce dehydration and slow down the degradation process of the mushrooms. Once again, as in other physicochemical parameters, there is a slightly better outcome at 9.3 °C than at 4 °C.

Despite the statistical analysis indicating that no significant differences can be found between samples, the literature supports the hypothesis that the coating slows down the weight loss of the mushrooms, as a study from Mohebbi et al. reached similar conclusions [28].

During the shelf life of the control samples, there is an observed increase in weight loss at 9.3 °C, which is probably due to the higher storage temperature. As the temperature rises, the dehydration process accelerates, resulting in greater weight loss over time. In contrast, the coated mushrooms exhibited a similar reduction in weight loss at both temperatures. This outcome can be attributed to the coating’s capacity to promote a more uniform weight loss even at elevated temperatures.

#### 3.3.3. Color

*A. bisporus* mushrooms change color throughout storage mainly due to browning [25]. This causes the usually white caps and flesh to become marked with brown spots, resulting from oxidation. Therefore, it is important to determine if this characteristic white changes during shelf life. L* (luminosity) is correlated with whiteness [25], and used as a tool to measure mushroom whiteness. As such, high L* values translate to whiter mushrooms. Table 5 summarizes the measurements of L* values for both control and coated mushrooms for the duration of the shelf-life study.

Overall, L* values decreased over time for both control and coated mushrooms at both temperatures tested. Browning is a fairly common phenomenon and this decrease in luminosity refers to its emergence. Mushrooms were less white at 9.3 °C (with decreases of 22.5% for control mushrooms and 14.8% for coated mushrooms) than at 4 °C (with decreases of 5.4% for control mushrooms and 1.9% for coated mushrooms). Coated mushrooms had lower luminosity variance (1.9% at 4 °C and 14.8% at 9.3 °C) than control mushrooms. These results indicate an improvement in mushroom preservation after coating application as it translated into less noticeable browning. Mohebi et al. [28] found that the application of plant-based coatings yielded similar results: coated mushrooms had less L* variation than control mushrooms. These results validate that the application of the developed coating improves mushroom preservation without negatively affecting the visual appeal.

Another important color change parameter is Δ*E*, where noticeable color differences between samples are quantified. Table 6 summarizes the information regarding calculated Δ*E* between control and coated mushrooms for all sampling times and tested temperatures.

From the calculated Δ*E*, it can be concluded that there is always a noticeable difference between control and coated mushrooms as the values are all >2.3 [36]. Differences between control and coated mushrooms were more noticeable for storage at 4 °C (Δ*E* = 19.0 at t_0_ and Δ*E* = 13.9 at t_14_), indicating that coated mushrooms were easily distinguishable from control mushrooms. However, at 9.3 °C, differences between treatments were less noticeable with time (Δ*E* = 10.5 at t_0_ and Δ*E* = 2.7 at t_14_). This finding, as discussed before, suggests a higher efficacy for the coating at higher storage temperatures. The fact that control mushrooms are easily distinguishable from coated mushrooms is expected as a result from the application of this type of edible coating [28] and, therefore, not necessarily a negative result. Results for color evaluation are very promising and indicate that the coating does not negatively affect consumers’ opinion on the mushroom.

#### 3.3.4. Texture

The texture is one of the organoleptic characteristics most affected during food storage [50]. It is expected that the application of a coating will reduce the variation of the evaluated parameter over time. As such, lower percentages of variation are the target. Button mushrooms are consumed whole (no parts are discarded upon preparation). Therefore, texture characterization was carried out on the stems and caps to ensure a better understanding of the effect of the coating. Table 7 presents the data for control and coated samples, showing percentage variations in different parameters after 14 days of storage for 4 °C and 9.3 °C.

At 4 °C, for *hardness*, control and coated mushrooms show less variation in the stem (−30% and −34%, respectively). Negative values for *hardness* in the stem indicate that it increased over time for both treatments. Additionally, positive values for *hardness* in the cap indicate that it lowered over time for both treatments. These observations can be explained by the loss in water content observed through weight loss (see Section 3.3.2). Stems, which are naturally more rigid, become stiff and caps lose volume and structure. For *cohesiveness*, control and coated mushrooms show less variation in the cap (32% and 3%, respectively). *Cohesiveness* is affected directly by water loss [51]. Smaller variations in *cohesiveness* indicate less water loss over time. Coated mushroom caps’ *cohesiveness* only varied by 3% after 14 days of storage. This suggests that, even though water loss is occurring, the applied coating can maintain the mushroom’s structural integrity.

At 9.3 °C, for *hardness*, control and coated mushrooms show less variation in the stem (7% and 32%, respectively). The positive values for *hardness* in both the cap and stem indicate that it lowered over time for both treatments. Unlike before, where *hardness* increased over time in the stems, *hardness* decreased over time in all mushroom parts for both treatments. This suggests a larger role of temperature in the structural integrity of the mushroom than before, seeing as weight loss was similar for both storage temperatures (see Section 3.3.2). For *cohesiveness*, control and coated mushrooms show less variation in the cap (−11% and −4%, respectively). The negative values observed indicate an overall increase in *cohesiveness* over time. Together with a decrease in *hardness*, this seems to indicate that for 9.3 °C, weight loss is less related to water loss than at 4 °C, as *cohesiveness* is directly affected by the presence of water.

Overall, both control and coated mushrooms had better texture-related parameters after 14 days of storage at 9.3 °C. This is concurrent with the present findings for other parameters (such as microbiological analysis) and brings forth the need to test the coating’s performance at higher storage temperatures.

## 4. Conclusions

After microbiological analysis, it was possible to conclude that the coating did not show antimicrobial effects, as various microorganisms were detected in both coated and control samples. The results indicate that storage temperature may play a more significant role in inhibiting microbial growth than the coating itself. Further research is needed to explore the influence of sterilization and aseptic work conditions, and also the migration flux of the microorganism from inside of the mushrooms that can influence the efficacy of the coating’s antimicrobial effects.

The pH values for coated and control mushrooms in the shelf-life study ranged from 6.35 to 6.75. Generally, non-coated samples had slightly lower pH. No significant differences were found, and pH levels remained within an acceptable range for both coated and control mushrooms.

Regarding the weight loss of samples, at 4 °C, both coated and control samples show similar weight losses. However, at 9.3 °C, the coated samples have lower weight losses, indicating the coating acts as a protective barrier against dehydration and degradation.

Throughout storage, *A. bisporus* mushrooms changed color due to browning and oxidation. L* values, which indicate whiteness, decreased over time for both coated and control mushrooms at 4 °C and 9.3 °C storage temperatures. Coated mushrooms showed less browning and had lower luminosity variance compared to control mushrooms. Overall, the coating did not negatively affect the mushroom’s consumer appeal.

The texture is a crucial organoleptic characteristic affected during food storage. At 4 °C, both treatments showed less variation in stem *hardness*, and caps lost volume and structure due to water loss. At 9.3 °C, both control and coated mushrooms exhibited better texture-related parameters, suggesting the coating’s positive impact on maintaining structural integrity at higher storage temperatures.

In summary, the impact of the coating on the physicochemical parameters was overall positive for the coating’s potential to preserve mushroom quality without hindering consumer appeal. Other influences on microbial growth inhibition and the protective barrier effect of the coating against dehydration and degradation should be explored in future approaches.

## Figures and Tables

**Table 1:** Obtained results of the microbiological analysis of the food matrices stored at a temperature of 4 °C, at different sampling times, t_0_ and t_14_, with and without coating. Counts are presented in log CFU/g. Counts higher than 300 CFU per plate were not considered.

		t_0_ Coated	t_0_ Control	t_14_ Coated	t_14_ Control
Total microorganisms	Detection	Yes	Yes	Yes	Yes
	Enumeration (log CFU/g)	Uncountable	5.85 ± 0.00	Uncountable	6.58 ± 0.00
Molds and yeasts	Detection	Yes	Yes	Yes	Yes
	Enumeration (log CFU/g)	Uncountable	6.46 ± 0.00	6.02 ± 0.00	4.18 ± 0.16
*Escherichia coli*	Detection	No	No	No	No
	Enumeration (log CFU/g)	n.a. *	n.a. *	n.a. *	n.a. *
Enterobacteriaceae	Detection	Yes	Yes	Yes	Yes
	Confirmation	No	No	No	No
	Enumeration (log CFU/g)	n.a. *	n.a. *	n.a. *	n.a. *
Coagulase-positive *Staphylococcus*	Detection	No	No	No	No
	Confirmation	n.a. *	n.a. *	n.a. *	n.a. *
	Enumeration (log CFU/g)	n.a. *	n.a. *	n.a. *	n.a. *
*Bacillus cereus*	Detection	No	No	No	Yes
	Confirmation	n.a. *	n.a. *	n.a. *	No
	Enumeration (log CFU/g)	n.a. *	n.a. *	n.a. *	n.a. *
*Pseudomonas*	Detection	Yes	Yes	Yes	Yes
	Confirmation	Yes	Yes	Yes	Yes
*Salmonella* spp.	Detection	No	No	No	No
	Confirmation	n.a. *	n.a. *	n.a. *	n.a. *
*Listeria* *monocytogenes*	Detection	No	Yes	No	Yes
	Confirmation	n.a. *	No	data	No

* Not applicable.

**Table 2:** Obtained results of the microbiological analysis of the food matrices stored at a temperature of 9.3 °C, at different sampling times, t_0_ and t_14_, with and without coating. Counts are presented in log CFU/g. Counts higher than 300 CFU per plate were not considered.

		t_0_ Coated	t_0_ Control	t_14_ Coated	t_14_ Control
Total microorganisms	Detection	Yes	Yes	Yes	Yes
	Enumeration (log CFU/g)	3.72 ± 0.89	2.93 ± 0.21	6.02 ± 0.35	5.55 ± 0.21
Molds and yeasts	Detection	Yes	Yes	Yes	Yes
	Enumeration (log CFU/g)	3.80 ± 0.50	4.00 ± 0.14	5.11 ± 0.10	4.60 ± 0.00
*Escherichia coli*	Detection	No	No	No	No
	Enumeration (log CFU/g)	n.a. *	n.a. *	n.a. *	n.a. *
Enterobacteriaceae	Detection	No	No	Yes	Yes
	Confirmation	n.a. *	n.a. *	Yes	Yes
	Enumeration (log CFU/g)	n.a. *	n.a. *	Uncountable	Uncountable
Coagulase-positive *Staphylococcus*	Detection	Yes	Yes	Yes	Yes
	Confirmation	No	No	No	No
	Enumeration (log CFU/g)	n.a. *	n.a. *	n.a. *	n.a. *
*Bacillus cereus*	Detection	No	No	No	No
	Confirmation	n.a. *	n.a. *	n.a. *	n.a. *
	Enumeration (log CFU/g)	n.a. *	n.a. *	n.a. *	n.a. *
*Pseudomonas*	Detection	Yes	Yes	Yes	Yes
	Confirmation	Yes	Yes	Yes	Yes
*Salmonella* spp.	Detection	No	No	No	No
	Confirmation	n.a. *	n.a. *	n.a. *	n.a. *
*Listeria* *monocytogenes*	Detection	No	No	Yes	Yes
	Confirmation	n.a. *	n.a. *	No	No

* Not applicable.

**Table 3:** pH values obtained for the samples without (control) and with coating, at temperatures of 4 °C and 9.3 °C, for both tested shelf-life times (t_0_ and t_14_).

Temperature	Sampling Time	Sample	pH
4 °C	t_0_	Control	6.60 ± 0.04
		Coated	6.39 ± 0.01
	t_14_	Control	6.70 ± 0.01
		Coated	6.68 ± 0.01
9.3 °C	t_0_	Control	6.35 ± 0.02
		Coated	6.47 ± 0.03
	t_14_	Control	6.75 ± 0.01
		Coated	6.60 ± 0.01

**Table 4:** Shelf-life weight loss values obtained for the samples without (control) and with coating at the two different storage temperatures: 4 °C and 9.3 °C.

Temperature	Sample	Weight Loss (%)
4 °C	Control	83.35 ± 2.42
	Coated	81.96 ± 3.57
9.3 °C	Control	87.43 ± 1.36
	Coated	81.90 ± 1.93

**Table 5:** Average values obtained for luminosity (L*) for mushrooms without (control) and with coating, at sampling times t_0_ and t_14_ and temperatures 4 °C and 9.3 °C.

Temperature	Sample	Time	L*	% Variance
4 °C	Control	t_0_	68.5 ± 17.6	5.4%
		t_14_	64.8 ± 2.7	
	Coated	t_0_	53.8 ± 9.8	1.9%
		t_14_	52.8 ± 9.4	
9.3 °C	Control	t_0_	62.7 ± 1.1	22.5%
		t_14_	48.6 ± 1.1	
	Coated	t_0_	60.1 ± 6.7	14.8%
		t_14_	51.2 ± 3.8	

**Table 6:** Calculation of ΔE* values for the comparison of samples without and with coating, at temperatures 4 °C and 9.3 °C, at both shelf-life times, t_0_ and t_14_, respectively.

Temperature	Time	Sample	L*	a*	b*	ΔE*
4 °C	t_0_	Control	68.5	18.8	97	19.0
		Coated	53.8	28.5	90	
	t	Control	64.8	33.6	93	13.9
		Coated	52.8	34.0	86	
9.3 °C	t_0_	Control	62.7	25.0	93	10.5
		Coated	60.1	34.4	89	
	t_14_	Control	48.6	36.6	87	2.7
		Coated	51.2	37.1	87	

**Table 7:** The percentage (%) of variation between t_0_ and t_14_ of texture characterization parameters of control and coated mushrooms stored at 4 °C and 9.3 °C.

Temp.	Sample		Weight	*Hardness*	*Cohesiveness*
4 °C	Control	Stem	27	−30	−61
		Cap	21	59	32
	Coated	Stem	17	−34	−32
		Cap	34	68	3
9.3 °C	Control	Stem	85	7	−50
		Cap	25	71	−11
	Coated	Stem	80	32	−18
		Cap	15	57	−4

## Data Availability

Data is contained within the article.

## Supplementary Materials

The following supporting information can be downloaded at: https://www.mdpi.com/article/10.3390/foods12163061/s1, Figure S1: Independent samples *t*-test of the total microorganisms’ microbiological analysis of the food matrices samples, without (control) and with coating, stored at temperature 4 °C; Figure S2: One Sample *t*-test of the total microorganisms’ microbiological analysis of the food matrices samples, without (control) and with coating, stored at temperature 4 °C; Figure S3: Test of Normality (Shapiro-Wilk) of the total microorganisms’ microbiological analysis of the food matrices samples, without (control) and with coating, stored at temperature 4 °C; Figure S4: Independent samples *t*-test of the total microorganisms’ microbiological analysis of the food matrices samples, without (control) and with coating, stored at temperature 9.3 °C; Figure S5: Test of Normality (Shapiro-Wilk) of the total microorganisms’ microbiological analysis of the food matrices samples, without (control) and with coating, stored at temperature 9.3 °C; Figure S6: Independent samples *t*-test of the molds and yeasts’ microbiological analysis of the food matrices samples, without (control) and with coating, stored at temperature 4 °C; Figure S7: One Sample *t*-test of the molds and yeasts’ microbiological analysis of the food matrices samples, without (control) and with coating, stored at temperature 4 °C; Figure S8: Test of Normality (Shapiro-Wilk) of the molds and yeasts’ microbiological analysis of the food matrices samples, without (control) and with coating, stored at temperature 4 °C; Figure S9: Independent samples *t*-test of the molds and yeasts’ microbiological analysis of the food matrices samples, without (control) and with coating, stored at temperature 9.3 °C; Figure S10: Test of Normality (Shapiro-Wilk) of the molds and yeasts’ microbiological analysis of the food matrices samples, without (control) and with coating, stored at temperature 9.3 °C; Figure S11: Independent samples *t*-test of the pH of the food matrices samples, without (control) and with coating, stored at temperature 4 °C; Figure S12: Test of Normality (Shapiro-Wilk) of the pH of the food matrices samples, without (control) and with coating, stored at temperature 4 °C; Figure S13: Independent samples *t*-test of the pH of the food matrices samples, without (control) and with coating, stored at temperature 9.3 °C; Figure S14: Test of Normality (Shapiro-Wilk) of the pH of the food matrices samples, without (control) and with coating, stored at temperature 9.3 °C; Figure S15: Independent samples *t*-test of the weight loss of the food matrices samples, without (control) and with coating, stored the two different storage temperatures: 4 °C and 9.3 °C; Figure S16: Test of Normality (Shapiro-Wilk) of the weight loss of the food matrices samples, without (control) and with coating, stored the two different storage temperatures: 4 °C and 9.3 °C; Figure S17: Independent samples *t*-test of the luminosity (*L**) of the food matrices samples, without (control) and with coating, stored at temperature 4 °C; Figure S18: Test of Normality (Shapiro-Wilk) of the luminosity (*L**) of the food matrices samples, without (control) and with coating, stored at temperature 4 °C; Figure S19: Independent samples *t*-test of the luminosity (*L**) of the food matrices samples, without (control) and with coating, stored at temperature 9.3 °C; Figure S20: Test of Normality (Shapiro-Wilk) of the luminosity (*L**) of the food matrices samples, without (control) and with coating, stored at temperature 9.3 °C; Figure S21: Independent samples *t*-test of the Δ*E** of the food matrices samples, without (control) and with coating, stored the two different storage temperatures: 4 °C and 9.3 °C; Figure S22: One Sample *t*-test of the Δ*E** of the food matrices samples, without (control) and with coating, stored the two different storage temperatures: 4 °C and 9.3 °C; Figure S23: Test of Normality (Shapiro-Wilk) of the Δ*E** of the food matrices samples, without (control) and with coating, stored the two different storage temperatures: 4 °C and 9.3 °C.
