# Supplementary material for: Microbiological Assessment of White Button Mushrooms with an Edible Film Coating
Source: Foods. 2023 Aug 15;12(16):3061. doi: 10.3390/foods12163061 (PMC10515315; doi:10.3390/foods12163061)
Supplement: Supplementary file 1 [file foods-12-03061-s001.zip › foods-2509571-supplementary.pdf]

foods MDPI

Supplementary Material

	Test	Statistic	df	p	Location Parameter	SE Difference	95% CI for Location Parameter	
							Lower	Upper
Control	Student	NaNa						
	Mann-Whitney	NaNa						

Figure S1. Independent samples T-Test of the total microorganisms' microbiological analysis of the food matrices samples, without (control) and with coating, stored at temperature 4 °C.

	t	df	p	Mean Difference	95% CI for Mean Difference	
					Lower	Upper
Control	37.816	5	< .001	6.212	5.790	6.635

Figure S2. One Sample T-Test of the total microorganisms' microbiological analysis of the food matrices samples, without (control) and with coating, stored at temperature 4 °C.

	W	p
Control	0.683	0.004

Figure S3. Test of Normality (Shapiro-Wilk) of the total microorganisms' microbiological analysis of the food matrices samples, without (control) and with coating, stored at temperature 4 °C.

	Test	Statistic	df	p	Location Parameter	SE Difference	95% CI for Location Parameter	
							Lower	Upper
Control	Student	−16.990	4	< .001	−2.666	0.157	−3.101	−2.230
	Mann-Whitney	0.000		0.077	−2.699		−3.000	−2.298
Coated	Student	−3.450	4	0.026	−2.029	0.588	−3.661	−0.396
	Mann-Whitney	0.000		0.077	−2.380		−2.969	−0.737

Figure S4. Independent samples T-Test of the total microorganisms' microbiological analysis of the food matrices samples, without (control) and with coating, stored at temperature 9.3 °C.

		W	p
Control	t0	0.923	0.463
	t14	0.750	< .001
Coated	t0	0.792	0.095
	t14	0.750	< .001

Figure S5. Test of Normality (Shapiro-Wilk) of the total microorganisms' microbiological analysis of the food matrices samples, without (control) and with coating, stored at temperature 9.3 °C.

	Test	Statistic	df	p	Location Parameter	SE Difference	95% CI for Location Parameter	
							Lower	Upper
Control	Student	NaNa						
	Mann-Whitney	NaNa						

Figure S6. Independent samples T-Test of the molds and yeasts' microbiological analysis of the food matrices samples, without (control) and with coating, stored at temperature 4 °C.

	t	df	p	Mean Difference	95% CI for Mean Difference	
					Lower	Upper
Control	10.396	5	< .001	5.319	4.004	6.634

Figure S7. One Sample T-Test of the molds and yeasts' microbiological analysis of the food matrices samples, without (control) and with coating, stored at temperature 4 °C.

	W	p
Control	0.733	0.014

Figure S8. Test of Normality (Shapiro-Wilk) of the molds and yeasts' microbiological analysis of the food matrices samples, without (control) and with coating, stored at temperature 4 °C.

	Test	Statistic	df	p	Location Parameter	SE Difference	95% CI for Location Parameter	
							Lower	Upper
Control	Student	NaN[a]						
	Mann-Whitney	NaN[a]						
Coated	Student	−4.589	4	0.010	−1.333	0.290	−2.139	−0.526
	Mann-Whitney	0.000		0.077	−1.574		−1.746	−0.678

Figure S9. Independent samples T-Test of the molds and yeasts' microbiological analysis of the food matrices samples, without (control) and with coating, stored at temperature 9.3 °C.

		W	p
Control	t0	0.750	< .001
	t14	NaN[a]	
Coated	t0	0.883	0.333
	t14	0.750	< .001

Figure S10. Test of Normality (Shapiro-Wilk) of the molds and yeasts' microbiological analysis of the food matrices samples, without (control) and with coating, stored at temperature 9.3 °C.

	Test	Statistic	df	p	Location Parameter	SE Difference	95% CI for Location Parameter	
							Lower	Upper
Control	Student	−4.143	4	0.014	−0.097	0.023	−0.161	−0.032
	Mann-Whitney	0.000		0.077	−0.100		−0.140	−0.050
Coated	Student	−44.000	4	< .001	−0.293	0.007	−0.312	−0.275
	Mann-Whitney	0.000		0.077	−0.290		−0.310	−0.280

Figure S11. Independent samples T-Test of the pH of the food matrices samples, without (control) and with coating, stored at temperature 4 °C.

		W	p
Control	t0	1.000	1.000
	t14	0.750	< .001
Coated	t0	0.750	< .001
	t14	1.000	1.000

Figure S12. Test of Normality (Shapiro-Wilk) of the pH of the food matrices samples, without (control) and with coating, stored at temperature 4 °C.

							95% CI for Location Parameter	
	Test	Statistic	df	p	Location Parameter	SE Difference	Lower	Upper
Control	Student	−31.537	4	< .001	−0.393	0.012	−0.428	−0.359
	Mann-Whitney	0.000		0.077	−0.390		−0.420	−0.370
Coated	Student	−8.528	4	0.001	−0.133	0.016	−0.177	−0.090
	Mann-Whitney	0.000		0.077	−0.140		−0.160	−0.100

Figure S13. Independent samples T-Test of the pH of the food matrices samples, without (control) and with coating, stored at temperature 9.3 °C.

		W	p
Control	t0	0.923	0.463
	t14	0.750	< .001
Coated	t0	0.893	0.363
	t14	0.750	< .001

Figure S14. Test of Normality (Shapiro-Wilk) of the pH of the food matrices samples, without (control) and with coating, stored at temperature 9.3 °C.

							95% CI for Location Parameter	
	Test	Statistic	df	p	Location Parameter	SE Difference	Lower	Upper
Control	Welch	−2.081	3.154	0.124	−4.087	1.964	−10.170	1.995
	Mann-Whitney	1.000		0.200	−4.611		−7.796	0.635
Coated	Welch	0.022	3.079	0.984	0.063	2.869	−8.936	9.061
	Mann-Whitney	5.000		1.000	0.096		−6.688	6.780

Figure S15. Independent samples T-Test of the weight loss of the food matrices samples, without (control) and with coating, stored the two different storage temperatures: 4°C and 9.3°C.

		W	p
Control	4.C	0.783	0.074
	9.3.C	0.909	0.415
Coated	4.C	1.000	0.993
	9.3.C	1.000	0.964

Figure S16. Test of Normality (Shapiro-Wilk) of the weight loss of the food matrices samples, without (control) and with coating, stored the two different storage temperatures: 4°C and 9.3°C.

	Test	Statistic	df	p	Location Parameter	SE Difference	95% CI for Location Parameter	
							Lower	Upper
Control	Welch	0.364	2.098	0.749	3.733	10.256	−38.477	45.943
	Mann-Whitney	6.000		0.700	5.300		−17.200	23.100
Coated	Welch	0.124	3.992	0.908	0.967	7.825	−20.777	22.710
	Mann-Whitney	4.000		1.000	−0.100		−19.700	16.500

Figure S17. Independent samples T-Test of the luminosity (L*) of the food matrices samples, without (control) and with coating, stored at temperature 4°C.

		W	p
Control	t0	0.989	0.795
	t14	0.964	0.637
Coated	t0	1.000	0.977
	t14	0.786	0.082

Figure S18. Test of Normality (Shapiro-Wilk) of the luminosity (L*) of the food matrices samples, without (control) and with coating, stored at temperature 4°C.

	W	df	p	Hodges-Lehmann Estimate	95% CI for Hodges-Lehmann Estimate	
					Lower	Upper
Control	9.000		0.100	14.400	12.300	16.600
Coated	8.000		0.200	9.200	−3.200	16.100

Figure S19. Independent samples T-Test of the luminosity (L*) of the food matrices samples, without (control) and with coating, stored at temperature 9.3°C.

		W	p
Control	t0	0.887	0.344
	t14	0.958	0.605
Coated	t0	0.851	0.242
	t14	0.815	0.150

Figure S20. Test of Normality (Shapiro-Wilk) of the luminosity (L*) of the food matrices samples, without (control) and with coating, stored at temperature 9.3°C.

	W	df	p	Hodges-Lehmann Estimate	95% CI for Hodges-Lehmann Estimate	
					Lower	Upper
Control	9.000		0.100	192.038	62.640	283.394
Coated	6.000		0.700	2.616	−9.447	12.776

Figure S21. Independent samples T-Test of the ΔE* of the food matrices samples, without (control) and with coating, stored the two different storage temperatures: 4°C and 9.3°C.

	t	df	p	Mean Difference	95% CI for Mean Difference Lower	Upper
Control	2.252	5	0.074	109.935	−15.566	235.436
Coated	4.165	5	0.009	9.059	3.468	14.650

Figure S22. One Sample T-Test of the ΔE* of the food matrices samples, without (control) and with coating, stored the two different storage temperatures: 4°C and 9.3°C.

		W	p
Control	9.3.C	0.991	0.821
	4.C	0.919	0.448
Coated	9.3.C	0.870	0.296
	4.C	0.949	0.565

Figure S23. Test of Normality (Shapiro-Wilk) of the ΔE* of the food matrices samples, without (control) and with coating, stored the two different storage temperatures: 4°C and 9.3°C.
